# Spatial Variation in the Educational Gradient of First Motherhood in Spain: Occurrence and Timing

**DOI:** 10.1002/psp.70106

**Published:** 2025-09-18

**Authors:** Manuel T. Valdés, Cristina Suero, Fabrizio Bernardi

**Affiliations:** 1 University of Vienna, Vienna, Austria; 2 University of Vienna - Wittgenstein Centre for Demography and Global Human Capital, University of Vienna, Vienna, Austria; 3 Universidad Nacional de Educación a Distancia (UNED), Madrid, Spain

**Keywords:** educational gradient, fertility timing, first child, geographical differences, Spain

## Abstract

Several studies have analyzed the relationship between educational attainment and fertility across countries, identifying significant differences. However, these works often overlook intra-country variation, which may play an important role in moderating that relationship. This study examines the heterogeneity in the educational gradient of fertility across Spain, a context characterized by lowest-low and latest-late fertility. Using data from the 2011 Spanish census, we reconstruct the reproductive history of 656,248 women aged 25–50, of whom 403,140 had become mothers. This large sample allows to analyze the educational gradient in first motherhood across Spanish provinces. To do so, we apply mixture cure models, which enable us to differentiate between the occurrence and the timing of the transition. The results reveal substantial regional variability in the educational gradient for both dimensions, although the association between the gradients in occurrence and timing is weak across space. We further find that cross-province variation in the educational gradient in the timing of the transition is largely driven by the heterogeneous behavior of non-university educated women, a pattern not observed for occurrence. Finally, we identify a negative association between the educational gradient and the province’s GDP per capita, particularly strong for the timing of the transition.

## 1 Introduction

The relationship between women’s educational attainment and fertility has been challenged in recent decades, with research revealing heterogeneous results based on individual and contextual factors (Vasireddy et al. 2023). Traditionally, the educational gradient of fertility was predominantly negative (Liefbroer and Corijn 1999), with highly educated women having fewer children. However, recent studies reveal considerable variation across time and space. For example, Jalovaara et al. (2019) reported a weakening of the negative educational gradient in cohort fertility in the Nordic countries, except Finland. Similarly, although childlessness remains more prevalent among highly educated women (Wood et al. 2014), they are more likely to progress to higher-order births once they become mothers (Compans et al. 2023; Wood et al. 2020). Notably, much of this literature relies on cross-country comparisons (Beaujouan et al. 2016; Van Bavel et al. 2018; Wood et al. 2014), while studies examining *within*-*country* variation remain scarce.

The analysis of the variation in fertility patterns at subnational levels is particularly relevant for population geography, where understanding the spatial dynamics of demographic behavior is a central concern (Entwisle 2007). Indeed, within-country variation in reproductive behavior, and particularly in the educational gradient of fertility, may reflect deeply rooted regional differences. Structural and institutional factors shaping the occurrence and timing of parenthood, such as labour market dynamics, access to early childcare, or prevailing social norms, vary substantially across regions within countries. As a result, attaining higher education, traditionally associated with a lower probability of entering motherhood and fertility postponement, may have varying effects on fertility behaviors depending on the specific local context (Nisén et al. 2021; Wood et al. 2020).

This study examines subnational variation in the educational gradient in the occurrence and timing of first motherhood in Spain, a country characterized by lowest-low and latest-late fertility (Lozano et al. 2024). Using microdata from the 2011 Spanish Population and Housing Census, we reconstruct the reproductive history of 656,248 women aged 25–50, including 403,140 who had become mothers by the time of the census. This large sample allows the examination of the educational gradient in first motherhood at the NUTS 3 level (Nomenclature of Territorial Units for Statistics,^1^ 52 geographical units). By retrieving the age at which women became mothers from coresident children, we examine not only the *quantum* but also the *tempo* of that transition.

We focus on first motherhood because, due to the tempoquantum effect, this transition has become essential for understanding fertility trends, particularly in low-fertility settings such as Spain (Lozano et al. 2024). As first motherhood is delayed, age-related declines in fecundity increasingly limit the possibility of having more children (Sobotka and Beaujouan 2018). Indeed, postponing pregnancy attempts is typically associated with a higher likelihood of experiencing infertility (Te Velde et al. 2012) and the underachievement of fertility intentions (Berrington and Pattaro 2014), ultimately affecting completed fertility (Beaujouan et al. 2023). At the same time, the educational gradient in the transition to the first child has also relevant implications for social reproduction. Highly educated women who do not become mothers cannot transmit their educational advantage to the next generation, partially offsetting the advantages that they provide to their children when they do become mothers (Song and Mare 2015). Thus, understanding the educational gradient in the occurrence and timing of the transition to the first child is essential for comprehending the broader fertility decline and the reproduction of social advantage.

With regard to the literature on population geography, our work makes a three-fold contribution. First, this article underscores the importance of geographic context in understanding a well-established relationship across demography, economics, and sociology, namely, the negative educational gradient in fertility. By conducting our analysis at the NUTS 3 level, we reveal substantial spatial heterogeneity, variation that remains obscured in conventional national-level analyses. This heterogeneity offers deeper insights into the mechanisms underlying educational gradients in fertility. In particular, we report a lower educational gradient in provinces with higher economic development, especially for the timing of the transition to first motherhood. Second, by examining separately the behavior of university and non-university-educated women, we found that the spatial variation in the educational gradient of fertility is primarily driven by the latter. Geography seems to matter fundamentally for less educated women, who tend to delay entering motherhood in wealthier provinces. Finally, by investigating separately the occurrence and timing of first motherhood, we observe that the educational gradients in both dimensions are only weakly associated across space, meaning that the educational gradient in the *tempo* of fertility in a specific local context cannot be deduced from the gradient observed in the *quantum*, or vice versa.

## 2 Theoretical Framework

### 2.1 Educational Attainment and the Transition to the First Child

The New Home Economics theory anticipates a negative relationship between educational attainment and fertility rates at the population level. As women attain higher levels of education and enter the labor force, the opportunity costs of motherhood rise significantly, leading to lower fertility rates (Becker 1993). The Gender Revolution Theory also posits a fertility decline following women’s increased participation in education and the labor market. According to this theory, the imbalance resulting from the double burden of work and family responsibilities influences women’s ability to childbearing. However, a second stage is predicted where gender equality extends to the household, with men sharing childcare and domestic responsibilities. This shift is expected to alleviate women’s double burden and eventually lead to a fertility rebound (Goldscheider et al. 2015), although no country has yet reached this level of gender equality to fully validate that prediction (Lappegård 2020). Nonetheless, if women increasingly outsource domestic work (Raz-Yurovich 2016) or public policies progressively facilitate the reconciliation between work and family (Wood and Neels 2019), the burdens of childbearing may be reduced for women, especially the highly educated, thereby narrowing the educational gradient in fertility.

At the individual level, previous research has examined the impact of education on the total number of children (Berrington et al. 2015; Requena 2021), as well as its effect on specific fertility transitions (Klesment et al. 2014; Wood et al. 2020). In this study, we focus on the progression to the first child, a crucial transition in contexts of delayed fertility that significantly shapes final fertility outcomes (Beaujouan et al. 2023). When analyzing the educational gradient in the transition to motherhood, it is essential to consider two dimensions: the *quantum* (whether the transition occurs) and the *tempo* (when the transition occurs) (Bernardi 2001).

Regarding the quantum, research has established a positive relationship between women’s educational attainment and permanent childlessness (Kreyenfeld and Konietzka 2017; Miettinen et al. 2015). The challenges women face in balancing work and family life constitute a key driver of the fertility decline observed in the European countries (McDonald 2013), which is especially relevant for highly educated women, as they usually experience a higher labor attachment (Wood and Neels 2017).

As for the timing of the transition, the delay of first motherhood usually varies by educational attainment (Gustafsson and Kalwij 2006), since highly educated women spend more time in formal education (Ní Bhrolcháin and Beaujouan 2012). The age at which women complete their education, in turn, influences the timing of other transitions to adulthood, such as entering the labor market or leaving the parental home (Billari and Liefbroer 2010), which finally affects the age at which women start attempting pregnancy (Billari et al. 2006). Furthermore, women with higher educational attainment tend to devote more time to advancing their careers and often wait until achieving greater job stability before having children (Kreyenfeld and Andersson 2014; Wood and Neels 2017).

In summary, previous research has identified not only an educational gradient in the likelihood of becoming mothers but also in the timing of this transition, with low-educated women entering motherhood more often and younger than their highly educated counterparts.

### 2.2 Geography and the Educational Gradient of Fertility

Place has been repeatedly shown to moderate fertility behavior, as key factors such as labor market conditions, family policies, and early childcare provision vary significantly across locations. Women are more likely to have children where labor markets offer greater security (Alderotti et al. 2021), work–family reconciliation policies are more generous (Fahlén 2013), and childcare facilities are widely available (Baizán 2009). Such spatial variation may, in turn, fuel spatial differences in the fertility behaviour of highly and less educated women. Consequentially, numerous studies have reported significant cross-national differences in fertility outcomes, including total fertility rates (TFRs) (Sobotka 2017), parity progression ratios (Zeman et al. 2018), period fertility (Greulich and Toulemon 2023), cohort fertility (Jalovaara et al. 2019), and age at first birth (Nathan and Pardo 2019).

Several works have also documented variation across countries in the educational gradient of fertility. For example, Wood et al. (2014) found that, across Europe, highly educated women were more likely to remain childless and had fewer children. However, their study also revealed considerable heterogeneity in the effect of education across countries. Relatedly, Van Bavel et al. (2018) showed that, in countries experiencing a baby boom, childlessness declined more sharply among women with above-primary education than among their lower-educated counterparts, and Beaujouan et al. (2016) observed that cross-national differences in childlessness were primarily driven by within-group changes rather than shifts in the educational composition of the population.

While these cross-country comparisons provide insightful evidence on the factors shaping fertility, they implicitly treat countries as internally homogenous units of analysis, thus overlooking the complexity within their frontiers. Since key drivers of country-level differences in fertility—such as labor market dynamics or childcare availability—often vary significantly across regions within the same country, significant internal variability is also expected.

The importance of the subnational context is well-established in fertility research. For example, Gray and Evans (2018) documented substantial variability in TFRs within Australia, with notably higher fertility levels in remote areas compared to major cities. In Germany, Goldstein and Kreyenfeld (2011) found highly persistent differentials between West and East Germany in both childlessness and the timing of fertility. Campisi et al. (2020), analyzing TFRs across European NUTS 3 regions, showed that regional fertility levels were shaped by GDP per capita, divorce rates, and the TFRs of neighboring regions. Similarly, Setz et al. (2025), using data at the NUTS 2 level, identified a spatial diffusion process in the prevalence of late fertility across neighboring regions.

Nevertheless, subnational studies on the educational gradient of fertility are uncommon. One notable exception is Nisén et al. (2021), who examined subnational variation in educational differences in cohort fertility at the NUTS 2 level across 15 European countries. Their findings revealed a high degree of within-country variation in the impact of education on cohort fertility rates, and showed that regions with higher economic development exhibited weaker educational gradients. Studying the Belgian case, Wood et al. (2020) found substantial spatial variation at the municipality level in the educational gradient in second birth hazards, which was connected to variation in childcare provision and median income. At a higher level of spatial disaggregation, Kuang et al. (2024) have recently reported significant differences in the educational gradient in cohort fertility within the UK. Our study joins this scant line of research and examines the cross-province variation (NUTS 3) in the educational gradient in the *quantum* and *tempo* of the transition to the first child in Spain.

### 2.3 The Spanish Case

#### 2.3.1 The Educational Gradient in Fertility in Spain

The Spanish context has been characterized by persistently low and late fertility for several decades. In 2011,^2^ the national TFR stood at 1.34 and the mean age at first birth was 30.1 years, both consistently far from the European Union average.^3^ While recent research has established no systematic connection between these two dimensions of fertility at the macro level, Spain emerges as an outlier (Beaujouan and Toulemon 2021), combining “lowest-low” and “latest-late” fertility levels.

These trends of declining fertility and pronounced postponement in Spain have been attributed to adverse economic conditions and persistent uncertainty in key domains relevant to family formation, particularly affecting younger generations (Bueno 2020). Specifically, difficulties in securing stable employment (Adsera 2006; Barbieri et al. 2015), challenges in accessing the housing market (Díaz-Fernández et al. 2019) and limited public support for families, such as insufficient childcare provision (Baizán 2009), have all been identified as key constraints on family formation. These factors influence fertility in two interrelated ways. First, they tend to delay the transition to parenthood by postponing preceding life-course events, such as leaving the parental home and forming a union (del Rey et al. 2022). Second, by postponing parenthood, they reduce the time available for having additional children, thereby affecting the quantum of fertility. Recent evidence indicates that delayed transitions to adulthood in Spain is associated with a higher likelihood of using assisted reproductive technologies, reflecting the challenges of conceiving at later ages (Suero et al. 2025).

As in many other countries, however, these trends depend heavily on women’s educational attainment. Spain has traditionally exhibited a negative educational gradient in fertility (Requena 2021), largely reflecting the lower likelihood of highly educated women becoming mothers (Esteve and Treviño 2019) and their tendency to postpone this transition (Vidal-Coso and Miret-Gamundi 2017). As a result, Spain stands out as one of the countries with the latest fertility among university-educated women in Europe (Rendall et al. 2010). However, once motherhood is initiated, highly educated women are more likely to proceed to subsequent births (Compans et al. 2023) and to achieve their fertility intentions (Lozano et al. 2024). These divergent patterns in the *quantum* and *tempo* of fertility are connected to the markedly different jobs, working conditions, and levels of economic security available to women with different levels of education (Bueno and García-Román 2021).

Importantly, factors shaping fertility intentions and transitions affect women differently depending on their educational attainment (Suero 2023). For instance, employment insecurity may be particularly detrimental for lower-educated women, especially in areas characterized by low institutional support for families (Fahlén 2013). This dynamic may contribute to substantial heterogeneity in the educational gradient of fertility, as public policies, unemployment rates, or the share of workers with permanent contracts differ widely across Spanish provinces. In regions where non-permanent employment is more prevalent, low-educated women may postpone entry into motherhood to a greater extent, potentially narrowing the gap with their highly educated counterparts. At the same time, highly educated women in a latest-late country such as Spain may have limited scope for further delays given the biological limits to postponement (Leridon and Slama 2008), constraining the potential for large spatial variability in their fertility behavior.

#### 2.3.2 Regional Heterogeneity

Spain is a highly heterogeneous country, marked by significant differences in economic structure, levels of wealth, and welfare provision, often aligned with linguistic and cultural distinctions, making it a compelling context for studies in population geography. It is politically and administratively decentralized, comprising 2 autonomous cities (Ceuta and Melilla) and 17 autonomous regions, which are further subdivided into 50 provinces.^4^ The characteristics of each province are closely tied to the autonomous region they belong to, as regions hold devolved competencies that allow them to implement their own policies across various domains. Nonetheless, substantial heterogeneity also exists within autonomous regions.

**Figure 1:**
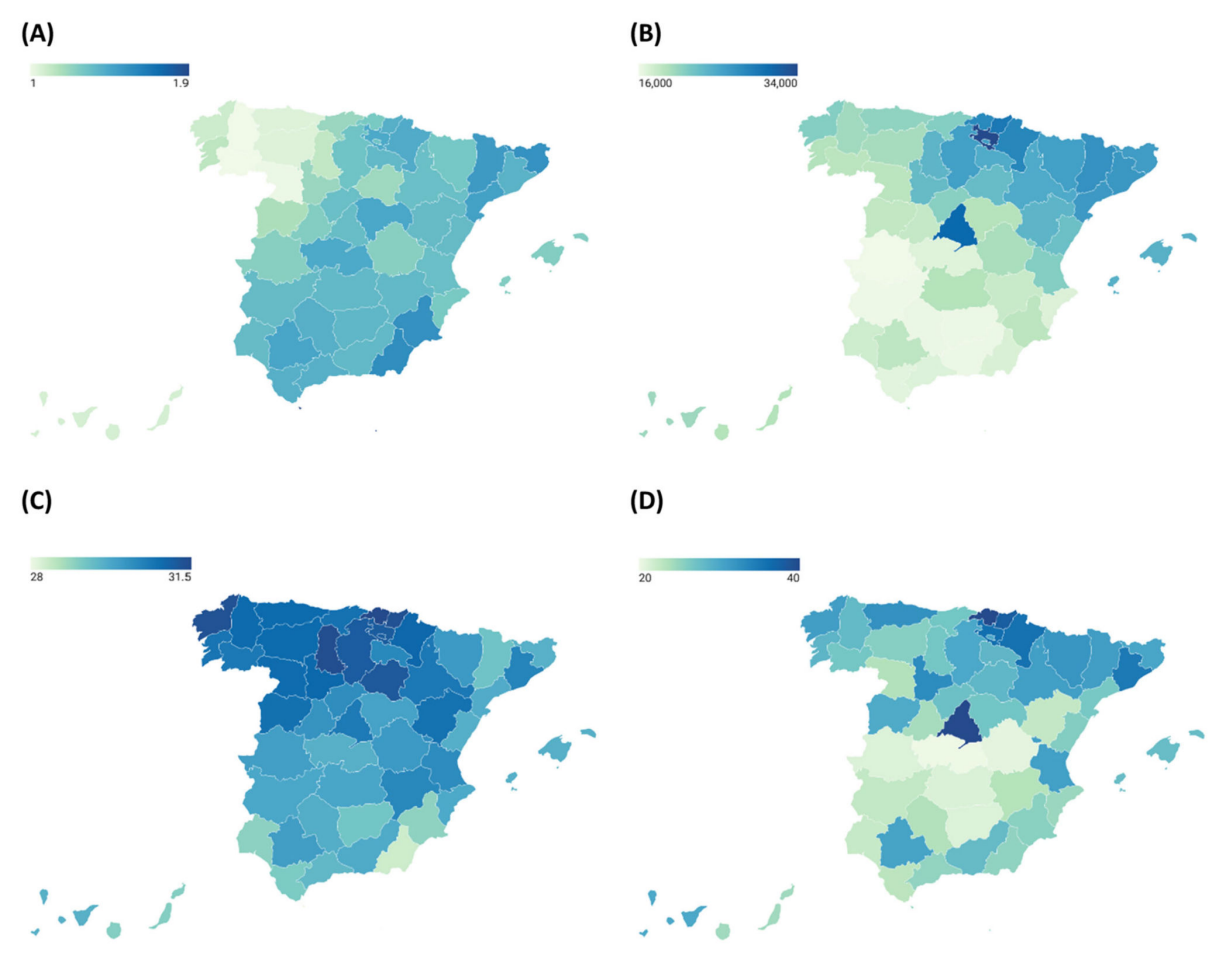
Variation across Spanish provinces in fertility behaviour and socioeconomic composition. All information is referenced to 2011 and sourced from the Spanish Statistical Office. Data on fertility is obtained from vital statistics. GDP_pc_ is obtained from Regional Accounting. The percentage of university-educated women aged 25–50 is obtained from the Spanish census. Exact figures are reported in Table S1A in the Appendix. (A) Total fertility rate. (B) GDP per capita. (C) Mean age at first birth. (D) Percentage of university-educated women (aged 25–50).

Figure 1 illustrates Spain’s internal heterogeneity at the provincial level using variables relevant to our analysis. Fertility rates are lower in the northwest (0.97 in Ourense), while southern provinces exhibit slightly higher rates (2.49 in Melilla, an autonomous city in North Africa). Regarding the timing of first motherhood, southern provinces tend to have younger mean ages at first birth (27.3 in Ceuta), whereas northern provinces report later ages, such as Vizcaya (31.5).

Macroeconomic conditions also vary substantially. Northern provinces report higher Gross Domestic Product per capita (GDP_pc_) than southern provinces, with Álava recording the highest GDP_pc_ (€34,200 per inhabitant) and Cáceres the lowest (€15,853 per inhabitant). These economic disparities not only reflect differences in purchasing power but are also closely linked to contrasting labour markets. In the north, labor markets feature a greater share of well-paying occupations and are less affected by unemployment (Ferrán Aranaz and Escot 2019). A similar north–south divide is evident in women’s educational attainment: provinces in the north have significantly higher proportions of university-educated women. The highest proportion is observed in Madrid (41.6%), while Toledo records the lowest (20.2%).

Against this backdrop of significant subnational heterogeneity in fertility rates, timing of first motherhood, and macroeconomic conditions, this study examines whether the educational gradient in the occurrence and timing of the transition to first motherhood varies across provinces.

## 3 Data and Methods

### 3.1 Source of Information

In many countries, family and fertility surveys provide insightful data for the analysis of fertility behavior. The main advantage of these surveys is that they offer detailed information on aspects such as fertility ideals and intentions, gender attitudes, or retrospective fertility histories. The major drawback is their limited size, which restricts the feasibility of analyses at high levels of spatial disaggregation. Alternatively, researchers might turn to register data, but most countries do not provide high-quality records of women’s reproductive histories. To overcome this limitation, this study reconstructs the reproductive history of a large sample of women using microdata from the 2011 Spanish Population and Housing Census. This data set includes information for around 10% of the total universe of people living in Spain in 2011: 4,107,465 observations, including 2,099,870 women.

Although the 2021 census data set is already available, we rely on the 2011 census because it includes information on the total number of children of each woman, which was not collected in 2021. This information allows us to assess the likelihood of the transition to the first child, but not its timing, as children’s birth dates are not reported. However, the age at first motherhood can be retrieved from co-resident children. To reconstruct fertility histories, we first identify women who became mothers using the total number of children. Next, we use kinship relationships within households to identify each mother’s coresident children. We then compare the number of co-resident children with each woman’s total children. If the two figures match, the mother lives with all their children and we can reconstruct her reproductive history, including birth order and the mother’s age at each birth. Conversely, if the total number of children exceeds the number of co-resident children, the mother does not live with all their children and it is not possible to reconstruct the complete reproductive history. For instance, if a mother has three children but lives with only two, it is not possible to know whether the non-resident child was the first, second, or third, preventing us from determining when she became a mother.

Therefore, our analytical sample includes only mothers living with all their children. While this represents a selected group of all mothers, this bias can be significantly reduced by focusing on women below a certain age. Indeed, while we can reconstruct the complete reproductive history for only 44.7% of all mothers over age 25,^5^ this figure increases to 90.7% for those aged 25–45 (Table 1). To strike a balance between sample size, minimizing selection bias, and allowing sufficient time for fertility trajectories to unfold, we limit our analysis to mothers aged 25–50. Subsequently, we include all childless women within these age limits. Our final analytical sample consists of 656,248 women aged 25–50, of whom 403,140 had become mothers by the time of the census (November 2011). Table S2A in the Appendix provides the distribution of the sample by province of birth and residence.

Table 2 presents the distribution of key variables in the original and analytical samples. Although we exclude 60,000 mothers who do not live with all their children, the differences between both samples are small. Women living with all their children tend to be slightly younger, somewhat better educated, marginally more likely to work as professionals, more often born in Spain, more likely single, and present a minimally lower average number of children. However, these differences are minor, suggesting that conditioning on living with all children does not introduce substantial bias. To further address potential concerns, we apply Entropy Balancing to generate a set of weights that balance the first three moments of the distribution of these variables (Hainmueller 2012). As shown in the third column of Table 2, all differences between the original and analytical samples nearly disappear after using EB weights. As a robustness check, we rerun the analysis using these weights to verify whether our conclusions are affected by conditioning on living with all children.

### 3.2 Methods

We employ survival analysis to examine our right-censored data on women’s transition to the first child. Specifically, we model the proportion of university- and non-university-educated women born in each province who do not complete this transition (*quantum*) and the age at which 50% of women who made the transition become mothers (*tempo*). Following recent demographic studies on fertility behaviour (Cukrowska-Torzewska and Grabowska 2023; Kreyenfeld et al. 2023; Puur et al. 2023), we use mixture cure modelling.

The cure model simultaneously estimates the proportion of the population who will experience the event and the duration until the event among those who will experience it (Lambert 2007). The model consists of two parts. The first estimates the probability of not making the transition under consideration, known as the cure fraction, which in our case corresponds to the complement of the Parity Progression Ratio. The second part estimates the timing of the transition specifically among those who made it. The equation of the model is:

$$S(t;x,\;y,\;z)=\pi (x)-(1-\pi (x))\;S_{b}(t;y,z)$$

where *S(t;x*,*y*,*z)* represents the survival function, *π(x)* stands for the proportion of women who will not make the transition, and *S*_*b*_*(t;y*,*z)* is the conditional survival function for susceptible individuals.

We use the identity link function to estimate the cure fraction:

π(x)=α+βx

where *x* is a vector of explanatory variables, and *β* is a vector of estimated coefficients.

For the survival function, we use a parametric model based on the lognormal distribution:

$$S_{b}(t;\;y,\;z)=1-\theta \left(\frac{\ln(t)-\mu }{\sigma }\right)=1-\theta \left(\frac{\ln(t)-y\beta_{y}}{z\beta_{z}}\right)$$

where *θ* is the cumulative distribution of the normal distribution, *t* is the time since the beginning of the spell, and *y* and *z* are vectors of explanatory variables.

For both parts of the model, we include a single predictor: women’s educational attainment, distinguishing those with a bachelor’s degree or higher (university-educated) and those with lower levels of education (non-university-educated). The model is estimated using the *strsmix* command in Stata 17 (Lambert 2007) and is adjusted separately for each province of birth, considering an additional geographical unit for women born abroad. By these means, we estimate the cure fraction and the median age at first birth for university- and non-university-educated women born in each Spanish province and abroad. However, two potential confounding factors warrant further discussion: (1) the different composition of the group of non-university-educated women across provinces and (2) internal migration.

First, spatial variation in the distinct fertility behaviour of university- and non-university-educated women could stem from differences across provinces in the composition of the non-university-educated group. In some provinces, this group includes more women with intermediate rather than low levels of education (see Table S3A in the Appendix). As a robustness check, we repeat the analyses comparing university-educated women with those who attained, at most, lower secondary education.

Second, we stratify our main analysis by province of birth to capture the context in which individuals grew up, as early-life socialization shapes both educational attainment and fertility behavior. Internal migration could, however, confound our findings if, for instance, career-oriented non-university-educated women born in poorer provinces systematically migrate to wealthier ones. Nonetheless, only 19.9% of native women in our sample experienced interprovincial mobility (i.e., the province of residence at the time of the census does not match the province of birth). While internal migration could still be problematic if it differed substantially across educational groups, this is not the case. University-educated women are slightly more likely to migrate, but the difference is modest: 23.1% among university-educated women and 18.4% among those without live in a province other than their province of birth.

Moreover, research on internal migration in Spain shows that internal mobility is low during childhood and adolescence, increases during the early 20s, and declines sharply in mid-working ages (Stillwell and Coll 2000). This pattern is particularly evident among the cohorts examined in this study, born between 1961 and 1986 (González-Leonardo et al. 2022). As a result, most individuals in our analytical sample would have spent their entire childhood and adolescence in their birth province, since parents rarely migrate after forming a family and children generally do not move until completing their education. Accordingly, the province of birth serves as a reliable proxy for the context in which the vast majority of individuals were raised.

Therefore, we consider internal migration unlikely to pose a major threat to our findings. To address any residual concerns, we conduct three robustness checks. First, we re-run the entire analysis using the province of residence at the time of the census instead of the province of birth. Second, we replicate the analysis only for women who reside in the same province they were born. Third, we use information on the birth province of co-residing children to construct an alternative measure of place: for mothers, the child’s province of birth, and for non-mothers, their own province of birth. This operationalization provides an intermediate measure of place in women’s mobility trajectories, capturing the local context in which mothers decided to have their first child. Consistent results across these analyses would provide strong evidence that internal mobility does not account for our findings.

## 4 Results

### 4.1 Cross-Province Variation in the Educational Gradient of the Transition to the First Child

**Figure 2:**
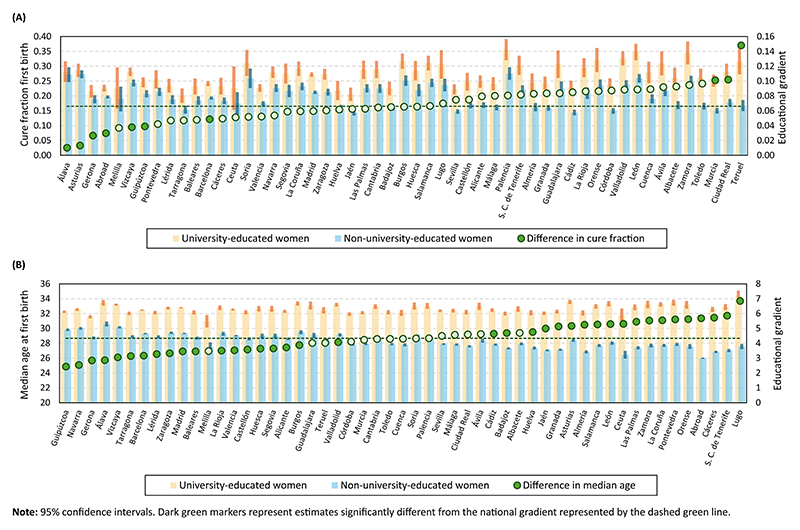
Predicted cure fraction and median age at first birth by women’s educational attainment in each province. 95% confidence intervals. Dark green markers represent estimates significantly different from the national gradient represented by the dashed green line. (A) Cure fraction. (B) Median age at first birth.

Figure 2 reports the results of our mixture cure models. The top panel displays the estimated cure fraction (i.e., the proportion of women that do not become mothers) by province of birth, while the bottom panel shows the estimated median age at first birth among those who made the transition. Yellow and blue bars indicate the estimates for university- and non-university-educated women, respectively, while green markers represent the educational gradient, defined as the difference between the two groups. Dark markers denote provinces where the 95% confidence interval of the educational gradient does not include the national-level estimate (horizontal dashed line), while light green markers indicate provinces where it does.

Figure 2 reveals substantial heterogeneity in the educational gradient in the transition to first motherhood across Spain. Regarding the occurrence of the transition, university-educated women are always more likely than their non-university-educated counterparts to remain childless. However, the magnitude of this difference varies widely, ranging from just 1 percentage point (pp) in Álava to 15 pp in Teruel. While these cases represent the extremes, the educational gradient in most provinces falls between 3 and 10 pp, still considerable variation.

However, in many provinces, the estimated differences are not precise enough to be statistically distinguishable from the national-level gradient.

The bottom panel of Figure 2 also reports a high degree of heterogeneity in the educational gradient in the median age at first birth. The difference is always positive, meaning that university-educated women become mothers later than their non-university-educated peers in all provinces. However, it ranges from 2.4 years in Guipúzcoa to 6.8 years in Lugo. Unlike the pattern observed for the occurrence, the educational gradient in the timing of the transition to first motherhood increases smoothly, without outliers at either extreme, and in most provinces it differs significantly from the national average.

Overall, these findings indicate that the women’s birthplace greatly shapes the effect of university education on the transition to the first child. However, the educational gradient is the result of the comparison of two quantities: the estimates for university- and non-university-educated women. Thus, the spatial variation in the educational gradient may stem from differences across provinces in the behavior of university-educated women, differences in the behavior of their non-university-educated counterparts, or both.

**Figure 3:**
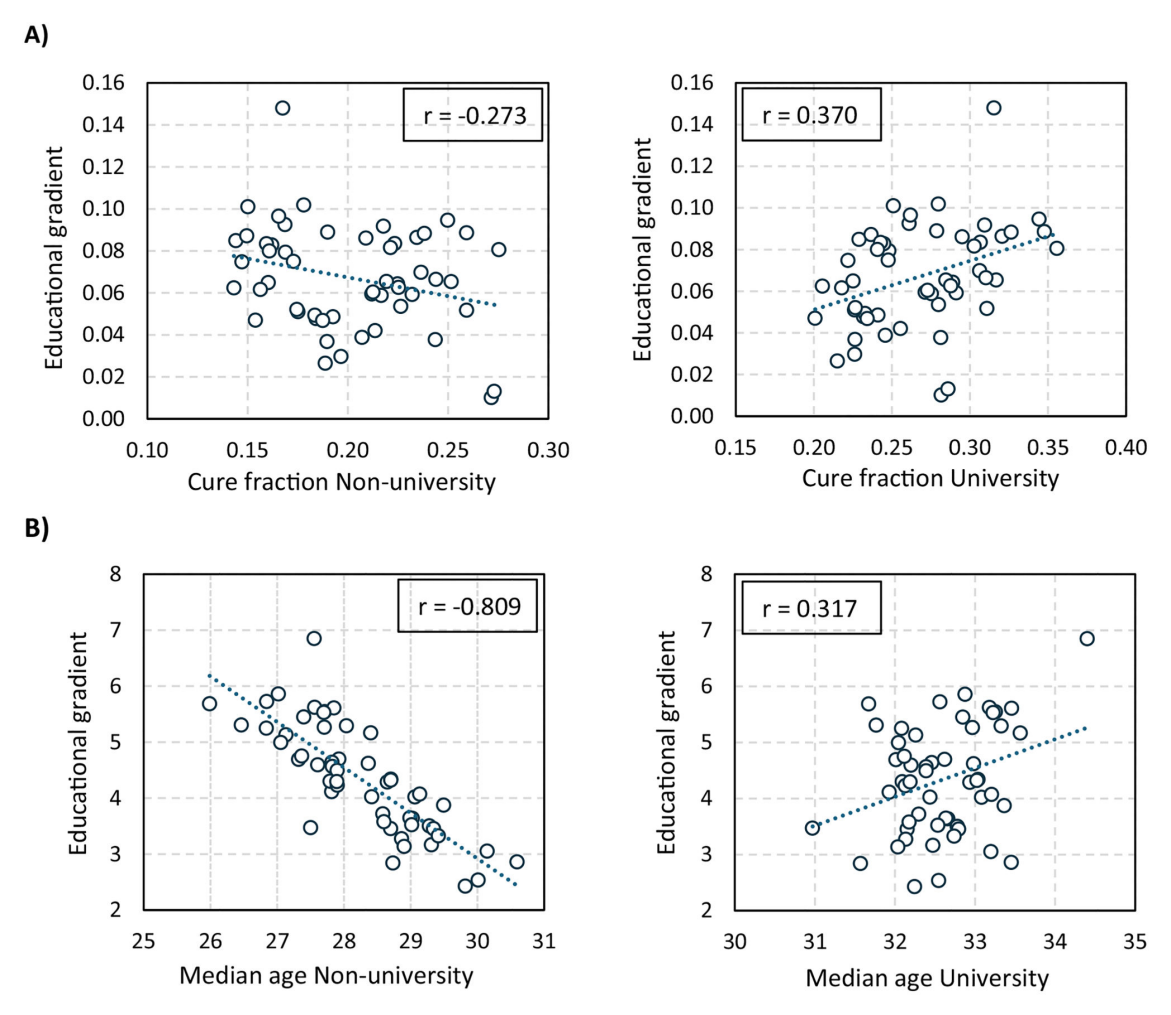
Effect of women’s education on the *tempo* and *quantum* of the transition to the first child against the behaviour of university and non-university-educated women. (A) Cure fraction. (B) Median age at first birth.

Figure 3 displays the association between the educational gradient in each province and the estimate of non-university-educated (left-side graphs) and university-educated (right-side graphs) women. Our findings indicate that the cross-province variation in the impact of women’s education on the median age at first birth (Panel B) is primarily driven by differences in the behavior of non-university-educated women (*r* = −0.81), whereas the correlation with the corresponding estimate for university-educated women is much weaker (*r* = 0.32). While university-educated women behave quite uniformly across Spain, the timing of first motherhood among non-university-educated women varies substantially, leading to the observed heterogeneity in the educational gradient.

No such pattern emerges for the occurrence of the first child (Panel A). The correlation between the educational gradient in the cure fraction and the estimates for university-educated women (*r* = 0.37) and non-university-educated women (*r* = −0.27) is moderate in both cases.

### 4.2 The Association Between the Educational Gradient in the Quantum and Tempo of Fertility

**Figure 4:**
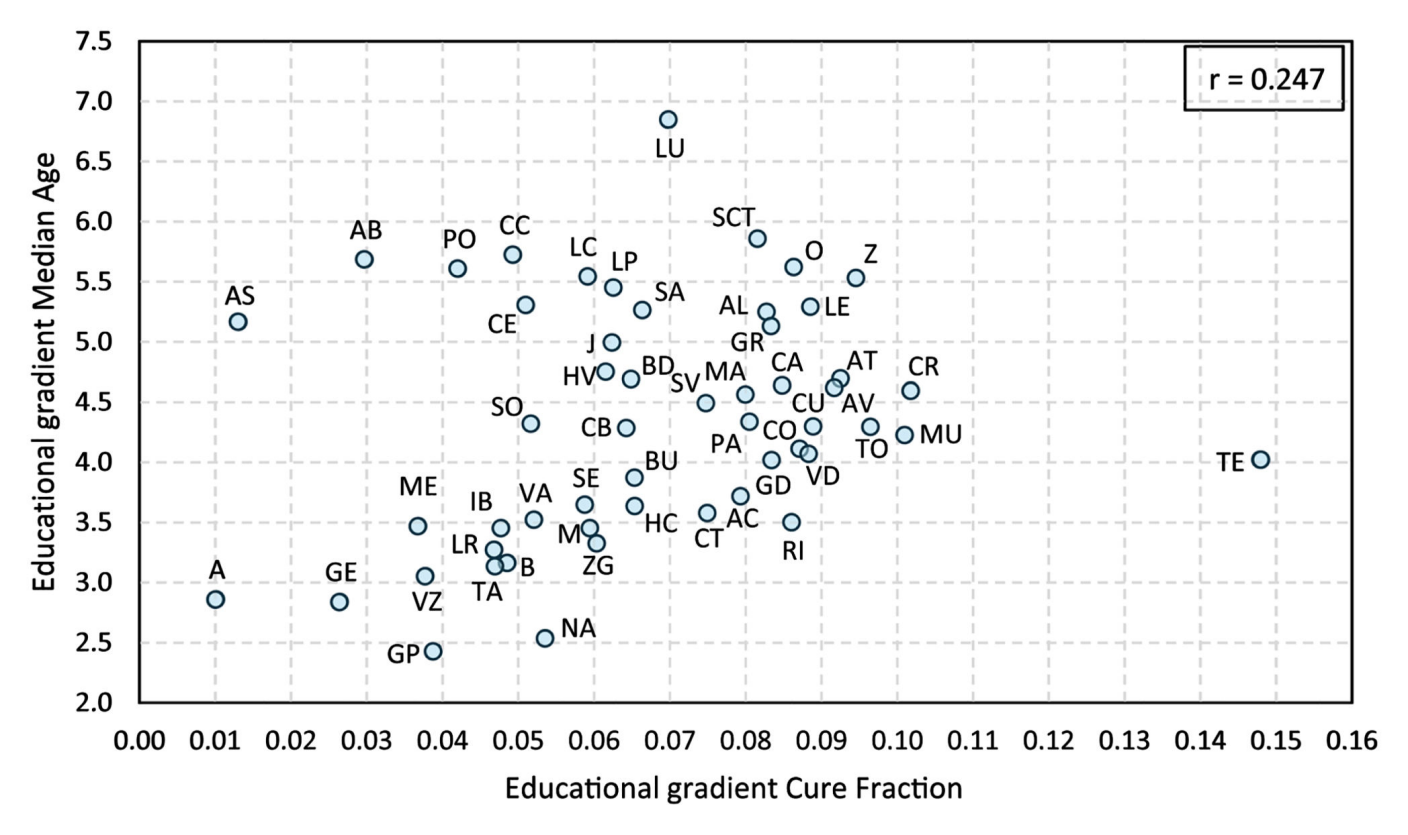
Association between the educational gradient in the occurrence (cure fraction) and timing (median age at first birth) of the transition to the first child across provinces. A, Álava; AT, Albacete; AC, Alicante; AL, Almería; AS, Asturias; AV, Ávila; BD, Badajoz; IB, Baleares; B, Barcelona; BU, Burgos; CC, Cáceres; CA, Cádiz; CB, Cantabria; CT, Castellón; CE, Ceuta; CR, Ciudad Real; CO, Córdoba; CU, Cuenca; GE, Gerona; GR, Granada; GD, Guadalajara; GP, Guipúzcoa; HV, Huelva; HC, Huesca; J, Jaén; LC, La Coruña; RI, La Rioja; LP, Las Palmas; LE, León; LR, Lérida; LU, Lugo; M, Madrid; MA, Málaga; ME, Melilla; MU, Murcia; NA, Navarra; O, Orense; PA, Palencia; PO, Pontevedra; SCT, S. C. de Tenerife; SA, Salamanca; SE, Segovia; SV, Sevilla; SO, Soria; TA, Tarragona; TE, Teruel; TO, Toledo; VA, Valencia; VD, Valladolid; VZ, Vizcaya; Z, Zamora; ZG, Zaragoza; AB, Abroad.

To assess whether provinces with a pronounced gradient in the occurrence of first motherhood also exhibit a correspondingly high gradient in the timing of the transition, Figure 4 presents the association across provinces between the two gradients. As shown, the relationship is weak (*r* = 0.247).^6^ For instance, Álava (A) and Asturias (AS) present similar low gradients in the probability of entering motherhood (0.010 and 0.013, respectively), yet markedly different gradients in the timing of the transition (2.9 and 5.2 years, respectively). Likewise, Zaragoza (Z) and Pontevedra (PO) exhibit similarly high educational gradients in the timing of the transition (5.5 and 5.6 years, respectively), but notably different gradients in the probability of entering motherhood (0.095 and 0.042, respectively). Therefore, the educational gradient in one dimension does not reliably predict the gradient in the other.

**Figure 5:**
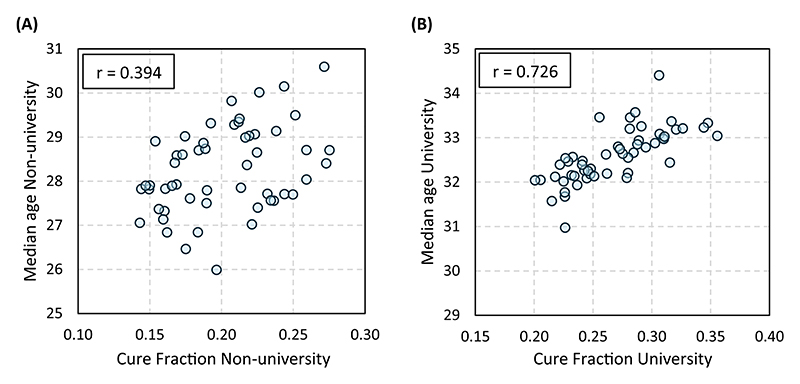
Association between the median age at first birth and the cure fraction across provinces. (A) Non-university-educated women. (B) University-educated women.

This weak correlation is compatible, however, with a strong *tempo*–*quantum* association within educational groups (Figure 5).^7^ The relationship is particularly strong among university-educated women (*r* = 0.73), as they postpone entry into motherhood to a larger extent and the connection between *tempo* and *quantum* intensifies at later ages (i.e., postponing 1 extra year reduces the probability of becoming a mother to a larger extent at 38 than at 28).

### 4.3 The Geographical Pattern in the Educational Gradient in the Transition to the First Child

**Figure 6:**
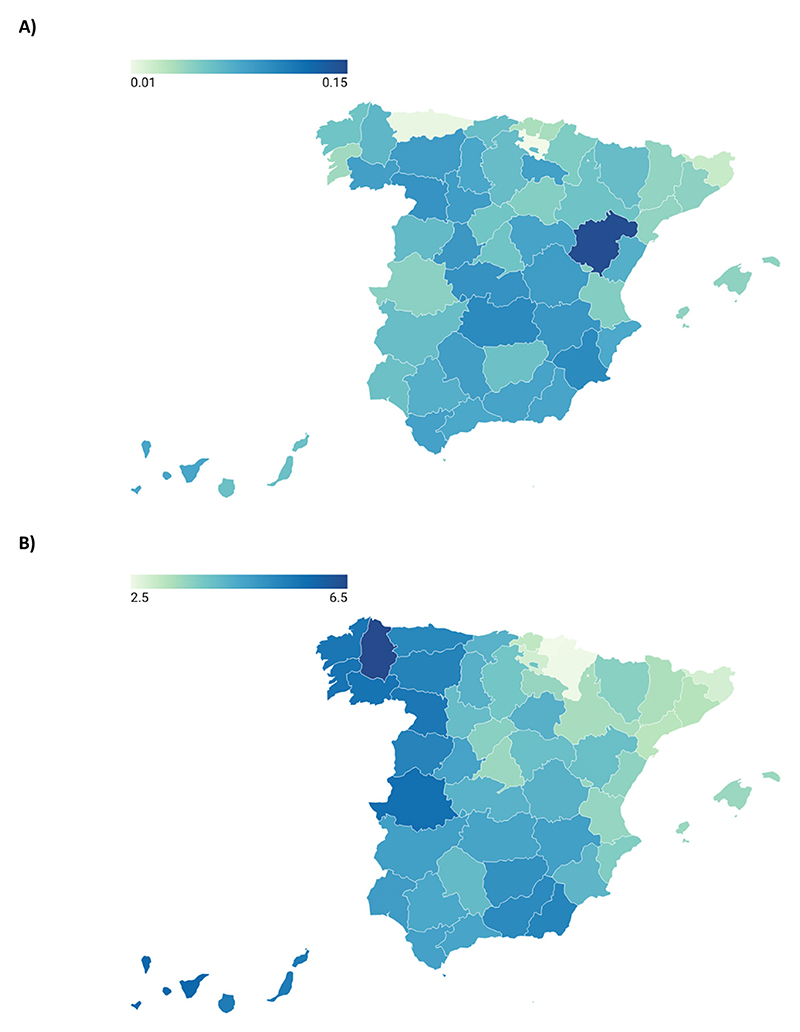
Map of the educational gradient in the occurrence and timing of the transition to the first child across Spanish provinces. (A) Educational gradient in the occurrence of first motherhood. (B) Educational gradient in timing of first motherhood.

Figure 6 maps the impact of attaining university education in the occurrence and timing of first motherhood. A distinct geographical pattern emerges for the educational gradient in the timing of the transition (Panel B), which is notably weaker in the north and the northeast of the country. The Basque provinces (Álava, Guipúzcoa, and Vizcaya) and Navarra exhibit particularly low gradients. Conversely, the educational gradient in the timing of first motherhood is notably higher in the south and, especially, in the northwest. The Galician provinces, Asturias, the west of Castilla y León (Salamanca, Palencia, and León), and the north of Extremadura (Badajoz) display the highest gradients.

No discernible geographical pattern is observed for the educational gradient in the occurrence of the transition to the first child (Panel A), which is consistent with the weak association between the educational gradient in *quantum* and *tempo* of first motherhood.

### 4.4 The Relationship Between the Educational Gradient in the Transition to the First Child and the Province’s Economic Development

**Figure 7:**
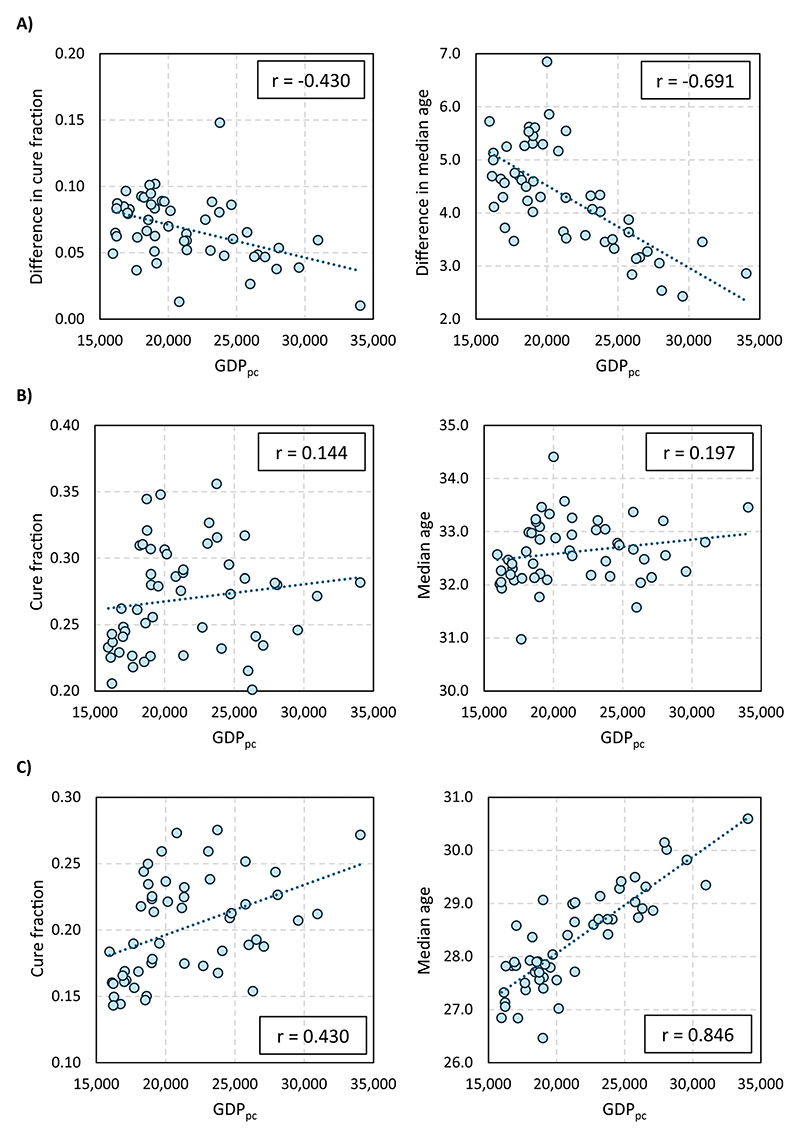
Relationship between the educational gradient in occurrence and timing of the transition to the first child and the GDP per capita of the province. GDP_pc_ data from year 2011. (A) Difference by educational attainment. (B) University-educated women. (C) Non-university-educated women.

Finally, Figure 7 examines the relationship between the educational gradient in the occurrence (left-hand graphs) and timing (right-hand graphs) of the transition to the first child and the economic development of the province of birth, measured by the GDP_pc_ in 2011.

As shown in the right-hand graph of Panel A, the impact of women’s education on the timing of the first child is strongly correlated with the province’s economic development (*r* = −0.69): the wealthier the province, the lower the difference in the timing of the progression to the first child between university- and non-university-educated women. Specifically, an increase in GDP_pc_ of €6.050—the interquartile range across provinces—would reduce the educational gradient in the median age at first birth by 0.9 years. Crucially, however, Panels B and C reveal that this relationship is entirely driven by the strong association between GDP_pc_ and the timing of fertility among non-university educated women (*r* = 0.85). In contrast, no meaningful association is observed with university-educated women (*r* = 0.20).

The left-hand graphs in Figure 5 tell a similar story for the *quantum* of fertility. Indeed, the difference in the probability of becoming mothers by educational attainment is lower in wealthier provinces, although the association is weaker (*r* = −0.43). Once again, this modest relationship is entirely driven by the behavior of non-university-educated women (*r* = 0.43), while GDP_pc_ has little association with the behavior of their university-educated counterparts (*r* = 0.14).

## 5 Robustness Checks

We have conducted several robustness checks to address potential limitations in our study (Table 3). First, our analytical sample excludes mothers not living with all their children. To mitigate this potential bias, we rerun the analysis using Entropy Balancing weights to correct imbalances in key variables. The results remain virtually unchanged, leading to the same conclusions as the main analysis. Thus, selection into our analytical sample does not seem to drive our findings.

Second, we grouped together low- and intermediate-educated women into the non-university-educated category to maximize sample size. However, this decision might be problematic if non-university-educated women in the north are mostly intermediate-educated, whereas in the South, they are pre-dominantly low-educated. If intermediate-educated women exhibit fertility behaviors more similar to university-educated women, this could partly explain our results. Therefore, we repeat the analysis comparing university-educated women with the subgroup of non-university-educated who attained, at most, lower secondary education. This more extreme comparison amplified the educational gradient, but the conclusions remained unchanged.

Finally, our findings can be sensitive to internal mobility. Reassuringly, our results remain highly consistent (1) when using the province of residence at the time of the census instead of the province of birth, (2) when using the province of birth but limit the analysis to women who reside in the same province they were born, and (3) when using the province of birth of the first child for mothers. This consistency suggests that internal mobility does not drive the patterns identified in our study.

## 6 Conclusions

This study examines variation across Spanish provinces (NUTS 3) in the educational gradient in the occurrence and timing of the transition to the first child. Using data from the 2011 Spanish census, our analysis yields five main findings. First, there is substantial variation across Spanish provinces in the educational gradient for both the occurrence and timing of first motherhood. Second, the association at the province level between the educational gradient in the occurrence and timing of the transition to the first child is weak. Third, a distinct geographical pattern emerges in the educational gradient of the timing of the transition, lower in the north and northeast of the country, and higher in the south and west. Fourth, there is a strong negative correlation between the educational gradient in the median age at first birth and the province’s economic development. Finally, this negative association is largely driven by non-university-educated women: while university-educated women display a highly uniform median age at first birth irrespective of the province’s GDP, their less educated counterparts significantly delay entry into motherhood when born in wealthier provinces.

Our findings align with Nisén et al. (2021), who also identified a negative relationship between the educational gradient in cohort fertility and GDP_pc_ across NUTS 2 regions in 15 European countries. Conversely, Wood et al. (2020) found that, in Belgium, the median income of a municipality increases the hazard of a second child for highly educated women but reduces it for medium and low-educated women. This divergence may stem from key differences in study design, since Wood et al. (2020) examined second births—where the educational gradient is typically positive—assessed only the occurrence of the transition and not its timing, and focused on Belgium, where neither childlessness nor delayed fertility are so extreme as in Spain.

Crucially, by not only examining the educational gradient but also assessing the specific behavior of university and non-university-educated women, this study offers important insights into the negative relationship between regional economic development and the educational gradient in fertility. Non-university-educated women tend to delay significantly entry into motherhood when born in wealthier provinces, thereby narrowing the gap with their university-educated counterparts. As discussed in the theoretical section, this finding may reflect the specific life conditions that less educated women face in these regions compared to poorer provinces, such as more demanding and time-consuming jobs (Yarger and Brauner-Otto 2024) or higher housing costs (Díaz-Fernández et al. 2019). Differences across provinces in social norms shaping ideals on age at entering motherhood may also play a role (Lazzari et al. 2024). In wealthier provinces, where university-educated women constitute a large share of the population, prevailing norms may mirror their reproductive behavior, encouraging delayed motherhood among less educated women despite having completed their education years earlier. In poorer provinces with fewer university-educated women, norms are likely to remain more traditional, prompting early entry into motherhood soon after the educational career is completed. In any case, the substantial variation in the timing of first motherhood among less educated women across Spain provides a compelling case for further investigation into the macro-level spatial determinants of fertility behavior.

Meanwhile, university-educated women behave quite uniformly across the country regardless of the province’s wealth. In a context of latest-late fertility such as Spain, where university-educated women significantly delay entry into motherhood, biological limits to postponement (Leridon and Slama 2008) may constrain the degree of spatial variation in their behavior (i.e., there is a ceiling effect), thereby limiting any potential association with the province’s wealth. By contrast, in countries where the transition to motherhood occurs earlier, spatial heterogeneity in the behavior of university-educated women may be more pronounced.

Overall, our work leads to several conclusions relevant to the broader literature on population geography. First, cross-country comparisons on the educational gradient in fertility often overlook a substantial degree of within-country heterogeneity, treating countries as units more homogeneous than they are. By showing the notable heterogeneity in the educational gradient of the *quantum* and *tempo* of the transition to first motherhood, our study contributes to a growing body of research that emphasizes the importance of descending from the national level into more fine-grained measures of space (Campisi et al. 2020; Nisén et al. 2021; Wood et al. 2020).

Second, geography seems to matter fundamentally for non-university-educated women, at least for the timing of the transition to the first child and within the Spanish context. We interpret space as a proxy for structural conditions, institutional arrangements, and cultural and normative frameworks that can influence reproductive choices. Low-educated women thus appear to be more sensitive to these contextual factors, particularly regarding the timing of first motherhood. This result connects with a recent strand of research concerned with the geography of inequalities initiated by the seminal work of Chetty et al. (2014). Just as the consequences of being raised in a socioeconomically disadvantaged family vary across geographic contexts, the impact of not attaining higher education on the timing and likelihood of first motherhood also depends on the specific setting in which women are born and raised.

Third, the data source allowed us to examine separately the spatial variation in the *quantum* and *tempo* of fertility. These analyses revealed that previous findings on the negative relationship between regional economic development and fertility behavior—based on measures of fertility that compound occurrence and timing—are largely driven by spatial variation in *tempo*. Interestingly, the educational gradients in the occurrence and timing of first motherhood are only weakly correlated across space, so that the magnitude of the gradient in one dimension cannot be inferred from the other. This result is compatible, nonetheless, with a strong association between quantum and tempo within educational groups.

As with any study, our research also has limitations. First, we rely on data from 2011, which may not fully capture recent dynamics in fertility behaviour, particularly the continued postponement of childbearing among university-educated women. However, by 2011 Spain was already among the countries with the latest mean age at first motherhood. Second, our analysis is conducted at the provincial level, which still encompasses internal heterogeneity. Nonetheless, moving to the NUTS 3 level represents a significant step forward in understanding the spatial variation in fertility behavior, as most existing research on subnational variation has typically been conducted at the NUTS 2 level. Third, our analytical sample excludes women not living with all their children, the composition of the non-university-educated group varies across Spain, and internal migration may affect results based on birthplace. Nonetheless, we provide robust evidence that none of these factors drive our conclusions. Future studies could build on our findings by incorporating detailed mobility trajectories to better understand how local contexts shape fertility decisions. Finally, while examining a single case allows us to control for factors constant across the country, it limits the generalizability of our findings beyond the fertility context of the Spanish case. Replicating this analysis in other contexts would further validate our interpretation of the results. Still, given the global trend of fertility postponement, the Spanish case offers valuable insights into a likely future for many countries.

To conclude, this study provides robust evidence of within-country heterogeneity in the educational gradient in the transition to the first child, underscores notable differences between occurrence and timing, and uncovers a strong and suggestive link between the size of the educational gradient in the timing of first motherhood and economic development.

## Figures and Tables

**Table 1:** Number of mothers in the original and analytical samples by age group.

	All mothers above 25	(25–65)	(25–60)	(25–55)	(25–50)	(25–45)
Original sample	1,210,230	814,846	705,435	592,548	462,962	327,428
Analytical sample	540,757	511,101	498,556	470,499	403,140	296,881
*%* retained	44.7%	62.7%	70.7%	79.4%	87.1%	90.7%

*Note*: Authors’ elaboration from the 2011 Spanish Population and Housing Census.

**Table 2:** Descriptive information about the samples (women aged 25–50).

	Original sample (1)	Analytical sample (2)	Analytical sample (weighted) (3)
Number of children			
Zero children	36.5%	38.6%	36.5%
One child	22.6%	23.4%	22.7%
Two children	33.0%	32.4%	32.9%
Three children	6.3%	5.0%	6.4%
Four children or more	1.6%	0.7%	1.5%
Age			
25−34 years old	33.2%	35.3%	33.2%
35−44 years old	43.7%	44.4%	43.7%
45−50 years old	23.2%	20.3%	23.2%
Educational attainment			
Non-university	69.2%	67.4%	69.2%
University	30.8%	32.6%	30.8%
Occupational status			
Directors and managers	2.2%	2.3%	2.2%
Professionals (STEM)	3.9%	4.5%	4.0%
Professionals (non-STEM)	19.1%	20.9%	19.1%
Employed—others	60.8%	60.3%	60.9%
Unemployed	13.9%	12.1%	13.9%
Country of birth			
Born abroad	11.2%	9.9%	11.2%
Born in Spain	88.8%	90.1%	88.8%
Civil status			
Single	32.9%	35.1%	32.9%
Married	59.1%	58.0%	59.1%
Widowed	1.2%	0.9%	1.1%
Separated	1.9%	1.6%	1.9%
Divorced	5.0%	4.4%	5.0%
Region			
Northeast	38.0%	38.3%	38.0%
Northwest	16.9%	16.8%	16.9%
Centre	18.9%	19.1%	18.9%
South	26.3%	25.9%	26.3%
*N*	729,570	656,248	656,248
% of the original	100%	89.9%	89.9%

*Note*: Authors’ elaboration from 2011 Spanish Population and Housing Census.

**Table 3:** Robustness tests for the relationship between GDP and the educational gradient in the occurrence and timing of the transition to the first child across provinces.

	Pearson coefficient with GDP _pc_						
	*Quantum*				*Tempo*		
	Non-university	University	Educational gradient		Non-university	University	Educational gradient
Main analysis	0.430	0.144	−0.441		0.846	0.197	−0.691
Entropy balancing	0.430	0.140	−0.445		0.846	0.193	−0.691
University vs. lower secondary	0.580	0.144	−0.567		0.741	0.197	−0.514
Province of residence	0.366	0.143	−0.273		0.847	0.201	−0.724
Non-movers	0.394	0.056	−0.355		0.842	0.212	−0.693
Province of birth of the child	0.066	−0.106	−0.251		0.833	0.238	−0.723

## Footnotes

1 The Nomenclature of Territorial Units for Statistics (NUTS) is a hierarchical classification system developed by the European Union to standardize regional divisions for statistical purposes across its member states. It is divided in three levels: NUTS 1 refer to major socioeconomic regions, NUTS 2 to basic regions, and NUTS 3 to small regions. Nevertheless, the implementation of the NUTS classification is adjusted to the specific context of each country.

2 The data are referred to 2011 as our empirical analysis exploits microdata for such year.

3 In 2011, the TFR in the EU-27 was 1.59. In 2013, the earliest year available in the data, the mean age at first birth in the EU-27 was 28.8 years. Information retrieved from: https://ec.europa.eu/eurostat/databrowser/view/demo_fin__custom_17333141/default/table? lang=en.

4 These 52 geographical units constitute the NUTS 3 level in Spain.

5 We only consider women above 25 to give them enough time to complete university education.

6 Teruel stands out as a highly deviant case regarding the educational gradient in the occurrence of first motherhood, which might affect our conclusions. Excluding Teruel, however, only rises the association to 0.300.

7 From a statistical perspective, there is nothing preventing two quantities from being highly correlated within two groups while the differences between groups are only weakly associated.

**Supporting Information**
Additional supporting information can be found online in the Supporting Information section. Appendix docx.

## References

[R1] Adsera A (2006). An Economic Analysis of the Gap Between Desired and Actual Fertility: The Case of Spain. Review of Economics of the Household.

[R2] Alderotti G, Vignoli D, Baccini M, Matysiak A (2021). Employment Instability and Fertility in Europe: A Meta-Analysis. Demography.

[R3] Baizán P (2009). Regional Child Care Availability and Fertility Decisions in Spain. Demographic Research.

[R4] Barbieri P, Bozzon R, Scherer S, Grotti R, Lugo M (2015). The Rise of a Latin Model? Family and Fertility Consequences of Employment Instability in Italy and Spain. European Societies.

[R5] Beaujouan E, Brzozowska Z, Zeman K (2016). The Limited Effect of Increasing Educational Attainment on Childlessness Trends in Twentieth-Century Europe, Women Born 1916–65. Population Studies.

[R6] Beaujouan É, Toulemon L (2021). European Countries With Delayed Childbearing Are Not Those With Lower Fertility. Genus.

[R7] Beaujouan E, Zeman K, Nathan M (2023). Delayed First Births and Completed Fertility Across the 1940–1969 Birth Cohorts. Demographic Research.

[R8] Becker GS (1993). A Treatrise on the Family Enlarged Edition.

[R9] Bernardi F (2001). Is It a Timing or a Probability Effect? Four Simulations and an Application of Transition Rate Models to the Analysis of Unemployment Exit. Quality and Quantity.

[R10] Berrington A, Pattaro S (2014). Educational Differences in Fertility Desires, Intentions and Behaviour: A Life Course Perspective. Advances in Life Course Research.

[R11] Berrington A, Stone J, Beaujouan E (2015). Educational Differences in Timing and Quantum of Childbearing in Britain: A Study of Cohorts Born 1940-1969. Demographic Research.

[R12] Billari FC, Liefbroer AC (2010). Towards a New Pattern of Transition to Adulthood?. Advances in Life Course Research.

[R13] Billari FC, Liefbroer AC, Philipov D (2006). The Postponement of Childbearing in Europe: Driving Forces and Implications. Vienna Yearbook of Population Research.

[R14] Bueno X (2020). Fertility Decisions in Transition: Young Adults’ Perceptions on Fertility Three Decades Apart in Spain. History of the Family.

[R15] Bueno X, García-Román J (2021). Rethinking Couples’ Fertility in Spain: Do Partners’ Relative Education, Employment, and Job Stability Matter?. European Sociological Review.

[R16] Campisi N, Kulu H, Mikolai J, Klüsener S, Myrskylä M (2020). Spatial Variation in Fertility Across Europe: Patterns and Determinants. Population, Space and Place.

[R17] Chetty R, Hendren N, Kline P, Saez E (2014). Where Is the Land of Opportunity? The Geography of Intergenerational Mobility in the United States. Source: The Quarterly Journal of Economics.

[R18] Compans M-C, Beaujouan E, Suero C (2023). Transitions to Second Birth and Birth Intervals in France and Spain: Time Squeeze or Social Norms?. Comparative Population Studies.

[R19] Cukrowska-Torzewska E, Grabowska M (2023). The Sex Preference for Children in Europe: Children’s Sex and the Probability and Timing of Births. Demographic Research.

[R20] del Rey A, Grande R, García-Gómez J (2022). Transiciones a la maternidad a través de las generaciones. Factores causales del nacimiento del primer hijo en España [Transitions to Motherhood Across Generations. Causal Factors in the Birth of the First Child in Spain]. Revista Española de Sociología.

[R21] Díaz-Fernández M, Llorente-Marrón M, Méndez-Rodríguez P (2019). Interrelation Between Births and the Housing Market: A Cointegration Analysis for the Spanish Case. Population, Space and Place.

[R22] Entwisle B (2007). Putting People Into Place. Demography.

[R23] Esteve A, Treviño R (2019). The Main Whys and Wherefores of Childlessness in Spain. Perspectives Demogràfiques.

[R24] Fahlén S (2013). Capabilities and Childbearing Intentions in Europe: The Association Between Work-Family Reconciliation Policies, Economic Uncertainties and Women’s Fertility Plans. European Societies.

[R25] Ferrán Aranaz M, Escot L (2019). Una propuesta metodológica para el análisis gráfico de series temporales regionales: una aplicación a las tasas de paro provinciales en España [A Methodological Approach for Regional Time Series’ Graphical Analysis: An Application to Unemployment Rates in Spanish Provinces]. Investigaciones Regionales-Journal of Regional Research.

[R26] Goldscheider F, Bernhardt E, Lappegård T (2015). The Gender Revolution: A Framework for Understanding Changing Family and Demographic Behavior. Population and Development Review.

[R27] Goldstein JR, Kreyenfeld M (2011). Has East Germany Over-taken West Germany? Recent Trends in Order-Specific Fertility. Population and Development Review.

[R28] González-Leonardo M, López-Gay A, Esteve A (2022). Inter-regional Migration of Human Capital in Spain. Regional Studies, Regional Science.

[R29] Gray E, Evans A (2018). Geographic Variation in Parity Progression in Australia. Population, Space and Place.

[R30] Greulich A, Toulemon L (2023). Measuring the Educational Gradient of Period Fertility in 28 European Countries: A New Approach Based on Parity-Specific Fertility Estimates. Demographic Research.

[R31] Gustafsson S, Kalwij A (2006). Education and Postponement of Maternity: Economic Analyses for Industrialized Countries.

[R32] Hainmueller J (2012). Entropy Balancing for Causal Effects: A Multivariate Reweighting Method to Produce Balanced Samples in Observational Studies. Political Analysis.

[R33] Jalovaara M, Neyer G, Andersson G (2019). Education, Gender, and Cohort Fertility in the Nordic Countries. European Journal of Population.

[R34] Klesment M, Puur A, Rahnu L, Sakkeus L (2014). Varying Association Between Education and Second Births in Europe: Comparative Analysis Based on the EU-SILC Data. Demographic Research.

[R35] Kreyenfeld M, Andersson G (2014). Socioeconomic Differences in the Unemployment and Fertility Nexus: Evidence From Denmark and Germany. Advances in Life Course Research.

[R36] Kreyenfeld M, Konietzka D (2017). Demographic Research Monographs.

[R37] Kreyenfeld M, Konietzka D, Lambert P, Ramos VJ (2023). Second Birth Fertility in Germany: Social Class, Gender, and the Role of Economic Uncertainty. European Journal of Population.

[R38] Kuang B, Berrington A, Christison S, Kulu H (2024). Educational Trends in Cohort Fertility by Birth Order: A Comparison of England and Wales, Scotland, and Northern Ireland. Demographic Research.

[R39] Lambert PC (2007). Modeling of the Cure Fraction in Survival Studies. Stata Journal.

[R40] Lappegård T (2020). Future Fertility Trends Are Shaped at the Intersection of Gender and Social Stratification. Vienna Yearbook of Population Research.

[R41] Lazzari E, Compans MC, Beaujouan E (2024). Change in the Perceived Reproductive Age Window and Delayed Fertility in Europe. Population Studies.

[R42] Leridon H, Slama R (2008). The Impact of a Decline in Fecundity and of Pregnancy Postponement on Final Number of Children and Demand for Assisted Reproduction Technology. Human Reproduction.

[R43] Liefbroer AC, Corijn M (1999). Who, What, Where, and When? Specifying the Impact of Educational Attainment and Labour Force Participation on Family Formation. European Journal of Population.

[R44] Lozano M, Esteve A, Boertien D, Mogi R, Cui Q (2024). Lowest Low Fertility in Spain: Insights From the 2018 Spanish Fertility Survey. Demographic Research.

[R45] McDonald P (2013). Societal Foundations for Explaining Fertility: Gender Equity. Demographic Research.

[R46] Miettinen A, Rotkirch A, Szalma I, Donno A, Tanturri M-L (2015). Increasing Childlessness in Europe: Time Trends and Country Differences. Families and Societies Working Paper Series.

[R47] Nathan M, Pardo I (2019). Fertility Postponement and Regional Patterns of Dispersion in Age at First Birth: Descriptive Findings and Interpretations. Comparative Population Studies.

[R48] Ní Bhrolcháin M, Beaujouan É (2012). Fertility Postponement Is Largely Due to Rising Educational Enrolment. Population Studies.

[R49] Nisén J, Klüsener S, Dahlberg J (2021). Educational Differences in Cohort Fertility Across Sub-National Regions in Europe. European Journal of Population.

[R50] Puur A, Abdullayev S, Klesment M, Gortfelder M (2023). Parental Leave and Fertility: Individual-Level Responses in the Tempo and Quantum of Second and Third Births. European Journal of Population.

[R51] Raz-Yurovich L (2016). Outsourcing of Housework and the Transition to a Second Birth in Germany. Population Research and Policy Review.

[R52] Rendall M, Aracil E, Bagavos C (2010). Increasingly Heterogeneous Ages at First Birth by Education in Southern European and Anglo-American Family-Policy Regimes: A Seven-Country Comparison by Birth Cohort. Population Studies.

[R53] Requena M (2021). Spain’s Persistent Negative Educational Gradient in Fertility. European Journal of Population.

[R54] Setz I, Compans MC, Beaujouan É (2025). The Diffusion of Late Fertility Across European Regions (2006-2018). Population, Space and Place.

[R55] Sobotka T (2017). Post-Transitional Fertility: The Role of Childbearing Postponement in Fuelling the Shift to Low and Unstable Fertility Levels. Journal of Biosocial Science.

[R56] Sobotka T, Beaujouan É, Stoop D (2018). Preventing Age Related Fertility Loss.

[R57] Song X, Mare RD (2015). Prospective Versus Retrospective Approaches to the Study of Intergenerational Social Mobility. Sociological Methods & Research.

[R58] Stillwell J, Coll AG (2000). Inter-Provincial Migration of the Spanish Workforce in 1988 and 1994. Regional Studies.

[R59] Suero C (2023). Gendered Division of Housework and Childcare and Women’s Intention to Have a Second Child in Spain. Genus.

[R60] Suero C, Compans M-C, Beaujouan E (2025). Delayed Transitions to Adulthood and Assisted Reproduction: A Study of Educational Differences in Spain. Advances in Life Course Research.

[R61] Te Velde E, Habbema D, Leridon H, Eijkemans M (2012). The Effect of Postponement of First Motherhood on Permanent Involuntary Childlessness and Total Fertility Rate in Six European Countries Since the 1970s. Human Reproduction.

[R62] Van Bavel J, Klesment M, Beaujouan E (2018). Seeding the Gender Revolution: Women’s Education and Cohort Fertility Among the Baby Boom Generations. Population Studies.

[R63] Vasireddy S, Berrington A, Kuang B, Kulu H (2023). Education and Fertility: A Review of Recent Research in Europe. Comparative Population Studies.

[R64] Vidal-Coso E, Miret-Gamundi P (2017). Characteristics of First-Time Parents in Spain Along the 21st Century. Revista Española de Investigaciones Sociológicas.

[R65] Wood J, Klüsener S, Neels K, Myrskylä M (2020). Shifting Links in the Relationship Between Education and Fertility. Population, Space and Place.

[R66] Wood J, Neels K (2017). First a Job, Then a Child? Subgroup Variation in Women’s Employment-Fertility Link. Advances in Life Course Research.

[R67] Wood J, Neels K (2019). Local Childcare Availability and Dual-Earner Fertility: Variation in Childcare Coverage and Birth Hazards Over Place and Time. European Journal of Population.

[R68] Wood J, Neels K, Kil T (2014). The Educational Gradient of Childlessness and Cohort Parity Progression in 14 Low Fertility Countries. Demographic Research.

[R69] Yarger J, Brauner-Otto SR (2024). Women’s Work Characteristics and Fertility Expectations. Population Research and Policy Review.

[R70] Zeman K, Beaujouan E, Brzozowska Z, Sobotka T (2018). Cohort Fertility Decline in Low Fertility Countries: Decomposition Using Parity Progression Ratios. Demographic Research.

